# Genetic Sorting of Subordinate Species in Grassland Modulated by Intraspecific Variation in Dominant Species

**DOI:** 10.1371/journal.pone.0091511

**Published:** 2014-03-17

**Authors:** Danny J. Gustafson, Charles Major, Dewitt Jones, John Synovec, Sara G. Baer, David J. Gibson

**Affiliations:** 1 Department of Biology, The Citadel, Charleston, South Carolina, United States of America; 2 Department of Plant Biology and Center for Ecology, Southern Illinois University Carbondale, Carbondale, Illinois, United States of America; The Australian National University, Australia

## Abstract

Genetic variation in a single species can have predictable and heritable effects on associated communities and ecosystem processes, however little is known about how genetic variation of a dominant species affects plant community assembly. We characterized the genetic structure of a dominant grass (*Sorghastrum nutans*) and two subordinate species (*Chamaecrista fasciculata*, *Silphium integrifolium*), during the third growing season in grassland communities established with genetically distinct (cultivated varieties or local ecotypes) seed sources of the dominant grasses. There were genetic differences between subordinate species growing in the cultivar versus local ecotype communities, indicating that intraspecific genetic variation in the dominant grasses affected the genetic composition of subordinate species during community assembly. A positive association between genetic diversity of *S. nutans*, *C. fasciculata*, and *S. integrifolium* and species diversity established the role of an intraspecific biotic filter during community assembly. Our results show that intraspecific variation in dominant species can significantly modulate the genetic composition of subordinate species.

## Introduction

Ecological change can direct evolutionary change on contemporary scales though ecological-evolutionary feedbacks, which may arise at different levels of biological organization, from genes to ecosystems. Individual genotype by environmental interactions direct phenotypic variation at the population level, whereas natural selection drives population dynamics as well as community interactions and ecosystem functions [1]–[6]. A new frontier in studying ecological-evolutionary (eco-evol) dynamics is determining the relative contribution of intraspecific genetic variation and genetic differentiation of a focal species on population, community, and ecosystem processes [7]. Although genetic variation in single species has been shown to have predictable and heritable effects on associated communities and ecosystems [2], [6], [8]–[12], this pattern is not universal [13].

Genetic variation within a species can be influenced by local biotic and abiotic conditions, resulting in a genetically distinct population (local ecotype) adapted to specific environmental conditions [14]–[16]. Many studies have documented local adaptation, identifying ecological and evolutionary factors that contribute to population genetic divergence [17]–[21]. Local ecotypes tend to outperform non-local ecotypes in their site of origin approximately 70% of the time, although evidence of divergent selection driving local adaptation (*sensu* Kawecki and Ebert [19]) was observed is less than half of the pair-wise comparisons [20], [21]. This intraspecific genetic variation can have ecological consequences on population dynamics, community structure, and ecosystem processes [2], [11], [12], [22]–[29]. Despite these advances in our understanding of community genetic dynamics, how intraspecific genetic variation in dominant species affects genetic structure of other species during plant community development has received less attention.

The objective of this study was to characterize the genetic structure of a dominant (*sensu* Grime [30]) warm season grass and two subordinate forb species in a community assembly experiment initiated with different seed sources of dominant species. Communities were sown with either cultivars or local ecotypes of dominant grass species (*Andropogon gerardii, Sorghastrum nutans*, and *Schizachyrium scoparium*) to establish field treatments with different genetic sources of grasses that typically comprise 80% or more of the biomass in tallgrass prairies. These grass species have been cultivated for improved forage production, erosion control, with rapid establishment, vigorous vegetative growth, high seed production and pest resistance [31]–[33]. Intraspecific genetic variation and differential ecological performance has been documented in these warm season grasses [16], [27], [34]–[40]. We quantified the genetic structure of subordinate species in response to intraspecific genetic variation of the dominant grasses. We hypothesized that the population source of the dominant grass species would differentially affect the genetic structure of subordinates in the developing communities. Based on previous research using the same seed sources, we expected genetic differences in the subordinate species to arise between plots sown with cultivar or local ecotype sources of the dominant grass species if intraspecific variation in these dominant species leads to genetic sorting of subordinate species during community assembly. Difference in the genetic structure (similarity or diversity) within subordinate species growing in the presence of the cultivar or local ecotype grasses treatments could represent an indirect interspecific genetic effect of dominant species genetic structure on community neighbors.

## Materials and Methods

### Field design and sampling

**Figure 1:**
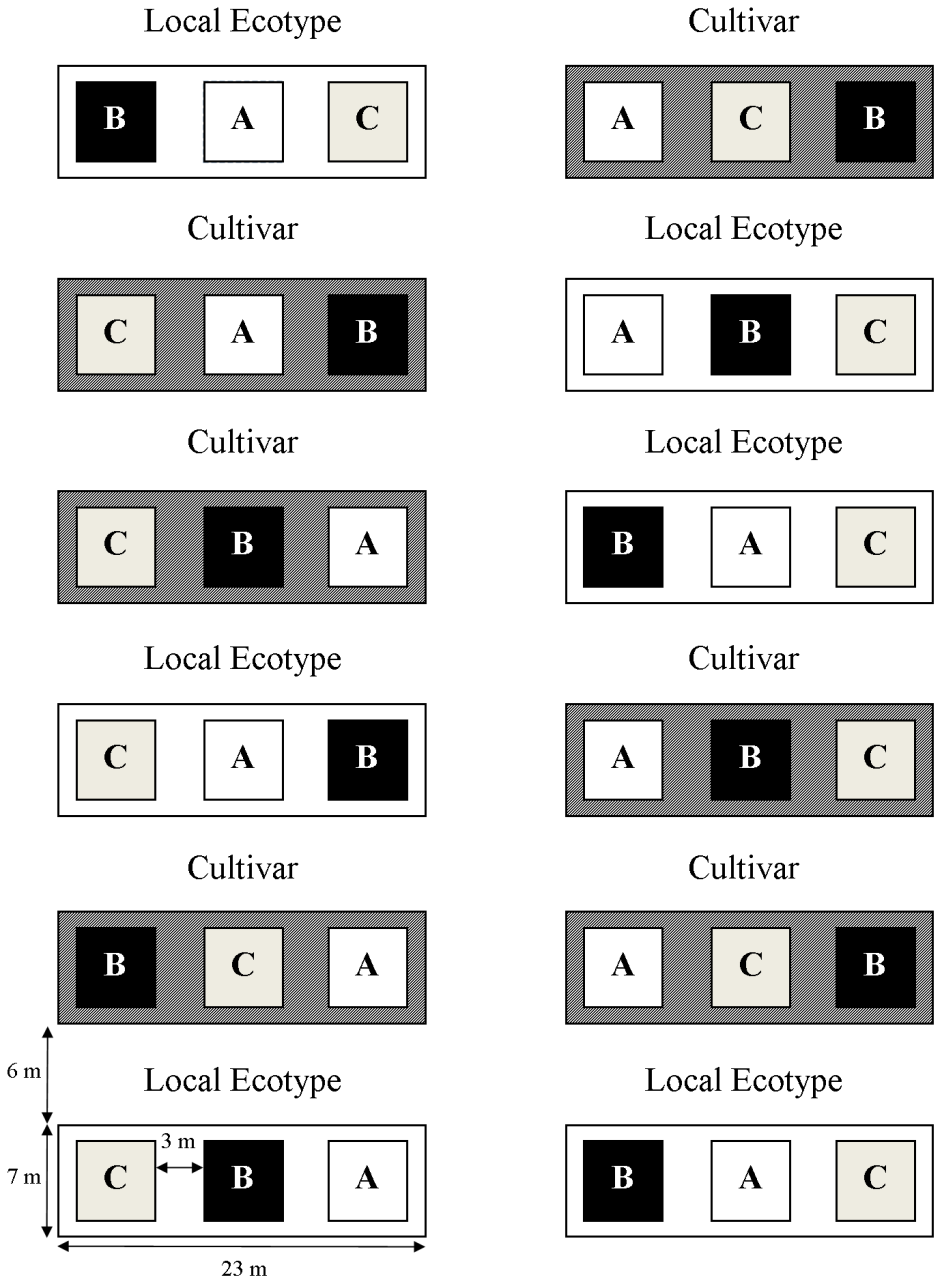
Field experimental design with three species pools (A,B,C) planted with local ecotype or cultivar grasses. The two subordinate species were sampled from their respective species pools (*Chamaecrista fasciculata*-A, *Silphium integrifolium*-B); the ‘Rumsey’ *Sorghastrum nutans* cultivar and local ecotype individuals were sampled across all three replicated species pools.

The field experiment contained 12 whole plots assigned to either cultivar or local ecotype source of dominant grasses (n = 6 per treatment) and three subplots seeded with different pools of subordinate species within each whole plot, resulting in six replicates of three different prairie communities (Fig. 1). We used ‘Rountree’ (*Andropogon gerardii* Vitman), ‘Aldous’ (*Schizachyrium scoparium* (Michx.) Nash), and ‘Rumsey’ (*Sorghastrum nutans* (L.) Nash) cultivars of each species per USDA recommendations for this region, based on land resource regions and plant hardiness zones [41]. Breeding methods of these cultivars were cross-pollination, increased field selection, and composite progeny of these accessions made after several generations of selection for seedling vigor, forage production, rust and lodging resistance [41]. The local ecotype seed for *A. gerardii*, *S. scoparium*, and *S. nutans* were hand collected from four remnant prairies within 75 km from the experimental field site [42]. Seeds of subordinate species were purchased from the most local native seed supplier (Hamilton Seed Co., Hamilton, MO, USA) [42]. The origins of the seeded subordinate species were not known; however, none were cultivated varieties and any potential variation within each subordinate species was assumed to be equally distributed among replicated subplots [42]. The dominant grasses were seeded at a rate of 300 live seeds m^−2^. The 15 other native species representing three unique species pools (A, B, and C) were each sown at a rate of 20 seeds m^−2^. Each species pool contained the same number of species representing four functional groups (C_4_ grasses, C_3_ grasses, legumes, and forbs) (Table S1). Six meter buffer areas between the whole plots were sown with two native prairie grasses, *Elymus canadensis* L. and *Bouteloua curtipendula* (Michx.) Torr. The focal annual and perennial species in this study, *Chamaecrista fasciculata* (Michx.) Greene (Fabaceae) and *Silphium integrifolium* Michx. (Asteraceae), were sown into species pools A and B, respectively.

The experiment was initiated in March 2006 at the Southern Illinois University Agronomy Research Center (37°41′N, 89°14′W). The formerly cultivated soil was classified as a fine-silty, mixed, superactive, mesic, Fragiaquic Hapludalf, with topsoil (0–0.25 m) comprised of silt loam and subsoil (0.25–1.30 m) of silt clay loam [25]. We sampled an increasingly dominant grass [42] and two subordinate species in the summer of 2008, corresponding to the third year of community assembly. Two to three newly expanded leaves were collected, of a single tiller or stem of a rooted plant, from up to 25 individuals for each of the three species from within each replicate dominant grass treatment, placed on ice, and stored at −20°C until genomic DNA was extracted. A total of 141 *Sorghastrum nutans* plants were sampled across all three species pools, whereas *C. fasciculata* and *S. integrifolium* were sampled from the species pools A and B, respectfully into which they had been sown. *Chamaecrista fasciculata* and *S. integrifolium* did not establish in each plot in which they were sown, hence a total of 203 *C. fasciculata* plants were sampled from 10 plots and a total of 135 *S. integrifolium* plants from 9 plots. Establishment of field plots, access, and field sampling efforts followed Southern Illinois University Agronomy Research Center policies and did not affect endangered or protected species.

To characterize the developing plant communities, we estimated percent cover of each species in 1 m^−2^ quadrats centrally located within each subplot in June and August of 2008. Only plots for which we had genetic data were included in the vegetation sampling. Because some early blooming species senesce by August, we used the common practice of calculating community metrics of Shannon's diversity (*H′*) and Simpson's diversity (*D*) using the maximum cover of each species from the late spring and summer surveys [43], as was used in the analyses of plant community dynamics in this experiment [44].

### Molecular Methods

Plant genomic DNA was extracted from approximately 0.5 g leaf material using a E.Z.N.A. plant DNA miniprep kit (Omega Bio-Tek, Norcross, Georgia, U.S.A.). Twenty five Inter-Simple Sequence Repeat (ISSR) primers were surveyed, with four polymorphic primers selected for each species (sequence, number of bands; *Chamaecrista fasciculata*, (CT)_8_RG, 5 bands; (CT)_8_T, 6 bands; (CA)_6_RY, 7 bands; (AC)_8_YA, 6 bands; *Silphium integrifolium*, (CA)_8_RT, 6 bands; (CA)_8_G, 5 bands; (AG)_8_YT, 6 bands; (AC)_8_YT, 5 bands; *Sorghastrum nutans*, (AG)_8_T, 10 bands; (GA)_8_C, 8 bands; (CT)_8_RC, 8 bands; (CA)_8_RC, 8 bands). ISSR polymerase chain reaction (PCR) protocol followed that of Wolfe *et al.* [45]; 94°C for 1 min 30 sec, 40 cycles of 94°C for 40 sec, 43°C for 45 sec, and 72°C for 1 min 30 sec, followed by a final extension at 72°C for 5 min. PCR profiles were visualized in 1.5% agarose gels and stained with ethidium bromide. Images were captured using a digital camera (Olympus C-4000 Zoom, Melville, NY), converted to a negative image, and fragment size was estimated based on a DNA marker (Benchtop pGEM, #G7521, Promega, Madison, WI). Fragment sizes were used to assign loci for each primer and bands were scored as diallelic for each locus (1 = band present, 0 = band absent). All *S. integrifolium*, *C. fasciculata*, and *S. nutans* individuals sampled in this study had unique ISSR DNA fingerprint profiles based on the four polymorphic primers and 22–34 loci profiles, respectively.

### Data Analysis

Percent polymorphic (*PP_ISSR_*) bands and Shannon's diversity (*H′_ISSR_*) were used to characterize the genetic diversity for all three species. We used multi-response permutation procedure (MRPP) to test the hypothesis that the genetic structure of the plants growing with cultivar grasses was different from the genetic structure of the same species growing with local ecotype grasses. A non-significant result indicates no difference in the genetic structure. Principal Coordinates Analysis (PCO) was used to investigate plot level genetic relationships of *C. fasciculata* and *S. integrifolium* using ISSR frequency data and relative Euclidean distance (PC-Ord, ver. 4.2, MjM Software Design, Gleneden Beach, Oregon, U.S.A.). Spearman correlations were used to test for associations between genetic diversity of all three species and tallgrass prairie community diversity, for the plots in which we have both genetic and community data, using SAS (SAS Enterprise Guide 4.3, SAS Institute Inc., Cary, NC).

## Results

**Figure 2:**
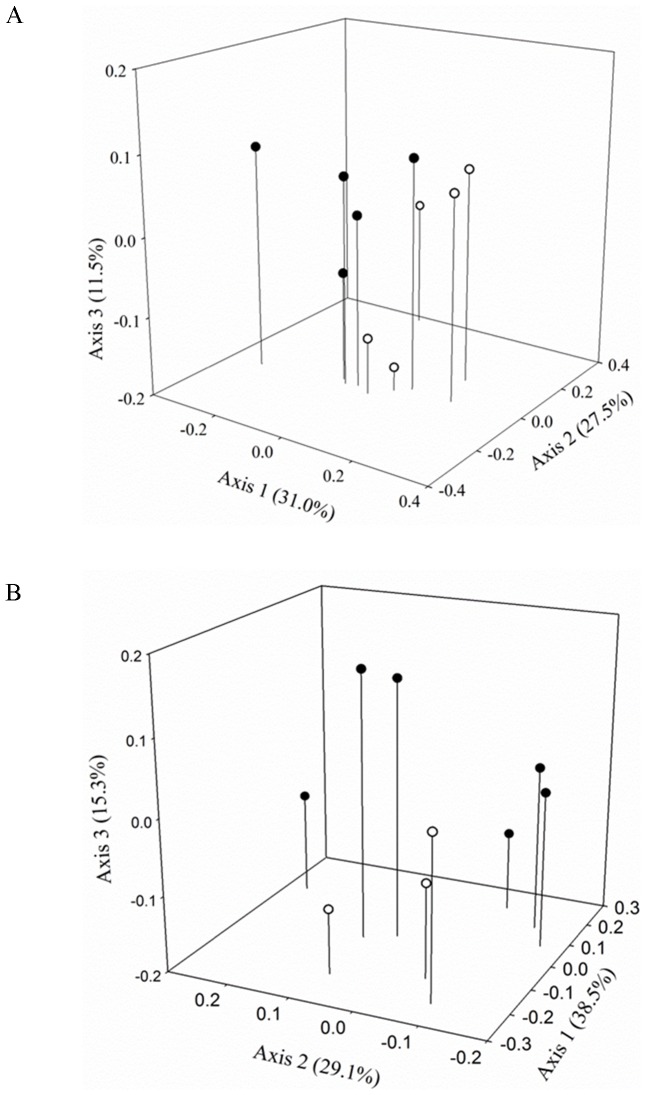
Principal coordinate analysis of *Chamaecrista fasciculata* and *Silphium integrifolium* based on ISSR band frequency data. Genetic relationships of *Chamaecrista fasciculata* (A) and *Silphium integrifolium* (B) growing in plots sown with cultivar (filled) and local ecotype (open) dominant grasses.

*Sorghastrum nutans* became the most abundant grass during assembly, with an average of 26.8 (±4.0) percentage cover compared to *A. gerardii* 7.2 (±1.2) and *S. scoparium* 6.6 (±1.5) during the third year of community assembly. Variation in genetic structure of the dominant grass was first reported in Baer et al. [42] to establish the efficacy of the seed source treatment. There was a significant difference in *S. nutans* genetic structure between the ‘Rumsey’ cultivar (T = −1.86, A = 0.004, *P*<0.05) and local ecotype source, which was expected given the different population sources. There were significant differences in genetic structure of the annual legume *C. fasciculata* (T = −5.51, A = 0.008, *P*<0.001) and perennial forb *S. integrifolium* (T = −2.53, A = 0.007, *P*<0.05) growing in the developing communities sown with the cultivar and local ecotype sources of the dominant grasses. Genetic relationships among plots based on ISSR frequency data also showed a general pattern of genetic differences between subordinate species growing in a matrix of cultivar dominant grasses relative to local ecotype sources (Fig. 2). Both *C. fasciculata* and *S. integrifolium* showed plot level separation according to dominant grass species source (cultivar or local ecotypes) with 70% and 82.9% of the variance explained in the first three PCO axes, respectfully.

**Figure 3:**
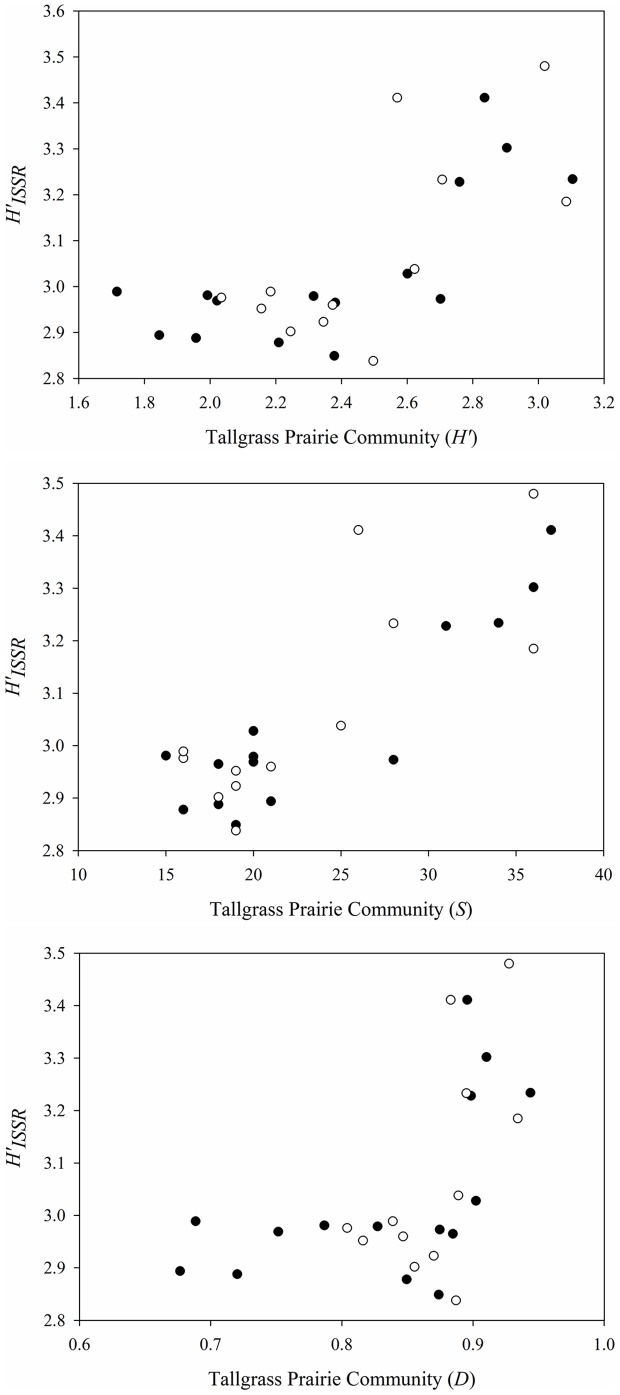
Genetic diversity by tallgrass prairie community structure associations. Scatter plots representing genetic diversity (*H′_ISSR_*) by tallgrass prairie community structure (*H′, S, D*) growing in plots sown with cultivar (filled) and local ecotypes (open) of the dominant grasses.

There were significant positive associations between ISSR genetic diversity of *S. nutans*, *C. fasciculata*, and *S. integrifolium* and plant community diversity (Fig. 3). In communities sown with cultivars of the grasses, percent polymorphic (*PP_ISSR_*) bands and Shannon's Diversity (*H′_ISSR_*) were positively correlated with tallgrass prairie community Shannon's Diversity (*H′*) and Simpson's Diversity (*D′*), however tallgrass prairie community species richness (*S*) was only positively correlated with Shannon's Diversity (*H′_ISSR_*) (Table 1). Tallgrass prairie community species richness (*S*) was positively correlated with *PP_ISSR_* and *H′_ISSR_*, and Shannon's Diversity (*H′*) was positively correlated with *H′ISSR* in the local ecotype grass communities.

**Table 1:** Spearman's Correlation Coefficient (*P*-value) between genetic diversity (*PP_ISSR_*, *H′_ISSR_*) and tallgrass prairie community diversity (*S, H′*, *D*) within cultivar and local ecotype dominant grass treatment.

		Tallgrass Prairie Community Diversity		
Grass Treatment	Genetic Diversity	*S*	*H′*	*D*
Cultivar	*PP_ISSR_*	0.38 (0.16)	0.55 (0.03)*	0.59 (0.02)*
	*H′_ISSR_*	0.62 (0.01)*	0.61 (0.02)*	0.60 (0.02)*
Local Ecotype	*PP_ISSR_*	0.72 (0.01)*	0.48 (0.11)	0.32 (0.30)
	*H′_ISSR_*	0.69 (0.01)*	0.62 (0.03)*	0.51 (0.09)

*P<0.05.

## Discussion

We tested a genetic assembly rule for dependent communities (*sensu* Bangert and Whitham), where intraspecific variation in the dominant warm season grasses differentially affected the genetic structure of two subordinate species in replicated assembling grassland communities established from seed [46]. Genetic, physiological, and competitive differences between warm-season perennial grasses developed as forage corps (cultivars) versus their non-selected local ecotype populations have been previously documented [25], [34], [37], [38], [47], [48]. The results of this study are novel in that they demonstrate genetic differences in two subordinate forbs of contrasting life history (i.e., the annual *C. fasciculata* and the perennial *S. integrifolium*) when grown with dominant perennial grasses from two different seed sources. For a grassland community dominated by C_4_ grasses, the *S. nutans* genotypes may provide a spatially varying biotic selection pressure through interspecific competitive interactions during community development. The genetic diversity of subordinate species can be filtered through competitive exclusion of subordinate species genotypes by dominant species during community assembly, which could explain the differences in the genetic structure we observed of *C. fasciculata* and *S. integrifolium* during the third year of community establishment. The species, and by extension their genotypes, that become established during the early stages of community assembly are the genetic individuals who will be available for driving subsequent population and community genetic dynamics. This biotic filtering of subordinate species genotypes by the dominant species genotypes is similar to the indirect abiotic filtering that can occur due to soil resource change or heterogeneity in grasslands [49].

There was a positive association between genetic diversity and community diversity, which may reflect local site characteristics positively influencing population genetic diversity and community diversity or possibly the direct effects of one level of diversity on another level of diversity during community assembly [50]. The notion that genotypic diversity of a dominant species provides a source of diversifying selection within the plant community is consistent with other grassland studies [51], [52]. Gibson *et al.* [44] and Baer *et al.* [42] demonstrated no strong effect of dominant grass source on community diversity and ecosystem processes for the first four years of community establishment from this field experiment. Similar diversity and functioning of communities sown with the different grass sources was attributed to the high productivity of subordinate species (i.e., a dilution effect), close proximity of the germplasm of the *S. nutans* cultivar to the local ecotype, and strong filtering of environment or site effects [42], [44]. Both studies acknowledge that there could be other (unmeasured) ecological consequences of using cultivars or long-term effects on community structure and ecosystem functioning, which could be exacerbated by variation in genetic structure and traits [6] of neighboring subordinate species. The differences in subordinate species genetic structure resulting from the two different dominant species sources may represent a future “hidden” effect of population source in community assembly.

This study captured the genetic patterns of one dominant and two subordinate species in replicated developing tallgrass prairie communities. If *S. nutans* and other sown dominant grasses continue to increase in cover and ANPP over time, their influence on community dynamics and biotic filtering could reduce genetic diversity through loss of individual genotypes [53]. If interspecific competitive outcomes are influenced by dominant species genotype by subordinate species interactions or specific genotype by genotype interactions, then biotic filtering could maintain or even promote genotypic and species diversity [50]. Using long-term experimental grassland plots, Whitlock *et al.* [23], [54] showed that individual genotypes achieved consistent abundance levels in genetically diverse communities. These genotypes exhibited substantial phenotypic variation driving genotype by environment interactions influencing competitive interactions. In the early stages of tallgrass prairie community assembly it is unclear whether genotype by genotypic interactions will promote community diversity through niche complementarity [24] or if genotypic specific dominance hierarchies will reduce community diversity through biotic filtering.

Quantifying the importance of intraspecific genetic variation relative to ecological factors is an ongoing challenge in community and ecosystem genetics [2], [6], [9], [27], [55], however it is also important to know how selection drives genetic divergence in communities across the landscape. In contrast to our community genetic findings, biotic filtering during early community assembly has had only limited effects on the community as a whole [52] and multiple aspects of ecosystem functioning [42]. This supports, in part, the role of intraspecific biotic filtering that we proposed [53], but not at all ecological scales. Ecological sorting may continue to drive genetic divergence of subordinate species between the communities established with grass cultivars and local ecotypes, respectively, or ecological factors may lead to community genetic convergence of subordinate genetic structure as these grassland communities continue to assemble (*sensu* Vellend and Geber [50]). Regardless, this study clearly demonstrates that genetic divergence can occur rapidly during community assembly and molecular markers can be used to quantify these population, community, and ecosystem genetic dynamics.

## Supporting Information

Table S1 **Tallgrass Prairie Community Species Pools (A, B, C).**
(DOCX)
