# Supplementary material for: Genetic Sorting of Subordinate Species in Grassland Modulated by Intraspecific Variation in Dominant Species
Source: PLoS One. 2014 Mar 17;9(3):e91511. doi: 10.1371/journal.pone.0091511 (PMC3956666; doi:10.1371/journal.pone.0091511)
Supplement: Table S1 — Tallgrass Prairie Community Species Pools (A, B, C). (DOCX) [file pone.0091511.s001.docx]

**Table S1.** **Tallgrass Prairie Community Species Pools (A, B, C).** Three different grassland communities were assigned to subplots within each whole plot seeded, with either cultivar or local ecotypes of dominant grasses. Genetic structure and diversity of *Chamaecrista fasciculata* and *Silphium integrifolium* were characterized within the context of species pools A and B; *Sorghastrum nutans* genetic structure was characterized across all three communities.

| **A** | **B** | **C** |
| --- | --- | --- |
| Forbs |  |  |
| *Asclepias tuberosa* | *Achillea millefolium* | *Aster oolentangiensis* |
| *Asclepias verticillata* | *Asclepias syriaca* | *Heliopsis helianthoides* |
| *Aster oblongifolius* | *Echinacea purpurea* | *Kuhnia eupatoroides* |
| *Callirhoe involucrata* | *Eupatorium altissimum* | *Monarda fistulosa* |
| *Delphinium virescens* | *Liatris pycnostachya* | *Penstemon digitalis* |
| *Oenothera macrocarpa* | *Oenothera biennis* | *Rudbeckia hirta* |
| *Ratibida pinnata* | *Ruellia humilis* | *Silphium laciniatum* |
| *Rosa arkansana* | *Silphium integrifolium* | *Solidago speciosa* |
| *Senecio plattensis* | *Solidago rigida* | *Vernonina fasciculata* |
| C_3_ Grass |  |  |
| *Elymus canadensis* | *Koeleria macrantha* | *Agrostis hyemalis* |
| C_4_ Grass |  |  |
| *Sporobolus heterolepis* | *Bouteloua curtipendula* | *Panicum virgatum* |
| Legumes |  |  |
| *Baptisia leucantha* | *Amorpha canescens* | *Astragalus canadensis* |
| *Chamaecrista fasciculata* | *Dalea candida* | *Baptisia leucophaea* |
| *Schrankia uncinata* | *Desmanthus illinoensis* | *Lespedeza capitata* |
| *Psoralea tenuiflora* | *Desmodium illinoense* | *Dalea purpurea* |
